# Editorial: Perspectives in the biotechnology of artificial insemination in ruminants

**DOI:** 10.3389/fvets.2024.1376794

**Published:** 2024-02-27

**Authors:** Stefan Gregore Ciornei, Omer Ucar, Graça Lopes, Mihai Cenariu

**Affiliations:** ^1^Faculty of Veterinary Medicine, Iasi University of Life Sciences, Iaşi, Romania; ^2^Milas Faculty of Veterinary Medicine, Muğla Sıtkı Koçman University, Muğla, Türkiye; ^3^School of Medicine and Biomedical Sciences (ICBAS), University of Porto, Porto, Portugal; ^4^Faculty of Veterinary Medicine, University of Agricultural Sciences and Veterinary Medicine of Cluj-Napoca, Cluj-Napoca, Romania

**Keywords:** artificial insemination, ruminants, reproductive biotechnologies, estrus, hormonal therapy

*Perspectives in the biotechnology of artificial insemination in ruminants*, a Research Topic hosted by Frontiers in Veterinary Science (Animal Reproduction - Theriogenology) was launched in November 2022. The aim was to provide an opportunity to different research groups to present their latest reports on various topics related to the biotechnology of artificial insemination (AI) in large ruminants, taking into consideration the need to enhance reproduction and production in a sector that plays a vital role in the global socioeconomic context. Ruminants provide a substantial source of income for farmers, specifically in developing countries, by supplying meat, milk, and other products. Assisted reproduction techniques, such as artificial insemination, enable breeders to improve the genetic traits of their herds, which leads to increased productivity, disease resistance, and welfare, in the advantage of both farmers and consumers. Thus, two original research papers, as well as a brief research report were published, together with a review, a mini-review and an opinion article.

Berean et al. investigated the economic implications and impact of gonadotropin-releasing hormone (GnRH) administration at the time of artificial insemination in cows, farmed extensively, in northern Romania. It is well known that a single dose of GnRH given at or just before the time of AI synchronizes the preovulatory LH surge and ovulation with AI (1) and is also related to higher progesterone levels after ovulation (2). Thus, this study aimed to assess the effect of GnRH analogs administration at the time of first, second, and third postpartum AI on the pregnancy rate, as well as its economical implication. The study involved both Romanian Brown and Romanian Spotted cattle and was conducted in small farms from northwestern Romania. GnRH administration improved the pregnancy rate after first and second AI by 12% and 18%, respectively. The cost of GnRH administration was ~49 Euros/pregnancy for the first insemination group and around 33 Euros/pregnancy for the second insemination group. No improvement of the pregnancy rate was observed after GnRH administration in cows at third insemination.

Borş et al. investigated the impact of mastitis on the reproductive activity and net present value (NPV) of high-yielding dairy cattle. Cytokines, such as tumor necrosis factor (TNF) are released into the bloodstream of cattle suffering from mastitis (3) which may induce prostaglandin F2α release and abnormal gonadotropin and GnRH production (4) and therefore negatively impact the reproductive activity of such animals. Thus, the objective of this study was to determine the net present value of mastitis in terms of milk loss, treatment costs, and impact on reproduction in a high-yielding dairy farm. Results showed that the negative impact of mastitis on NPV is mostly due to the cost of milk loss (US$14,439.4/farm/year) and treatments (US$4,380/farm/year), while the economic loss associated with poor reproductive performance (US$3,577/farm/year) represents only an additional cost, despite the impairment of reproductive function (i.e. mastitic high-yielding dairy cows had a significantly lower conception rate).

Ciornei and Roşca provided an upgrade of the fixed-time artificial insemination (FTAI) protocol in Romanian buffaloes. As the number of local buffaloes in Romania is constantly decreasing (5), the application of reproductive biotechnologies in these animals remains limited. Thus, increasing the efficacy of AI could represent an opportunity to improve their reproductive output. This study was carried out on buffalo heifers, that were synchronized using the Ovsynch protocol and divided into 2 groups. Classical FTAI was performed in the control group, while in the experimental group, efficiency of the ovarian response was assessed by ultrasound examination, and deep ipsilateral intrauterine FTAI with sexed semen was performed, only in heifers that had a dominant follicle larger than 0.9 cm. The conception rate (CR) was 63.6% in the experimental group and was statistically higher (*P* < 0.05) than in the control group (30%). Thus, by implementing the upgraded protocol, a greater conception rate was obtained, which may be beneficial to buffalo populations if expanded to a larger scale.

Gupta et al. reviewed the pathogenesis and immunotherapy of bovine reproductive immunoinfertility. Although infertility remains one of the biggest issues in cattle breeding nowadays (6), immunoinfertility is still unexplained and therefore misdiagnosed (7). This review provides important data about the causes and mechanisms by which blockage of receptor sites by antibodies formed against hormones, sperm and ova can lead to various reproductive disorders, such as anovulation or delayed ovulation, sperm immobilization, fertilization failure, prolonged uterine involution, extended calving interval, prolonged post-partum estrus and reduced conception rates. It also presents the therapeutic options of immunoinfertility, including sexual abstinence, reproductive biotechnologies (*in vitro* fertilization, gamete intrafallopian tube transfer, intracytoplasmic sperm injection) as well as the use of immunomodulators.

Andrei et al. reviewed the semen separation techniques in buffaloes. Since reproductive technologies applied in the buffalo sector are permanently facing several challenges, the use of high-quality semen has to be addressed for the optimization of FTAI protocols. Computer assisted sperm analysis (CASA) systems are able to provide an accurate and objective insight into the fertility parameters of males (8), but semen separation techniques are needed to eliminate low-quality spermatozoa, unwanted cells, and bioactive particles, quickly and economically, especially when assisted reproductive technologies are used. This paper reviews the current methods of semen separation, such as the density gradient centrifugation method, the swim-up method, the filter separation (sephadex gel and glass wool), as well as the magnetic-activated cell sorting.

Last but not least, Filho et al. presented an interesting opinion paper on the usefulness of models and simulators for training practical bovine AI skills. Such instruments are very useful at the initial stages of bovine AI training, as they can reduce the harmful effects of training on a living animal and contribute to the wellbeing of cows used for education. Nevertheless, new developments are needed in order to attain a broader implementation of the 3Rs, due to current high initial investment and maintenance costs.

In summary, the results of the above mentioned studies and reviews represent an important and useful database for researchers, scholars and practitioners involved in ruminant medicine, with an emphasis on reproduction and assisted reproductive technologies.

## Author contributions

SC: Conceptualization, Writing—original draft. OU: Validation, Writing—review & editing. GL: Validation, Writing—review & editing. MC: Conceptualization, Supervision, Writing—review & editing.
